# Comparison of Long-Term Antibody Titers in Calves Treated with Different Conjunctival and Subcutaneous *Brucella abortus* S19 Vaccines

**DOI:** 10.3390/ani15020212

**Published:** 2025-01-14

**Authors:** Ali Uslu, Zafer Sayın, Aslı Balevi, Yasin Gulcu, Fırat Ergen, Islam Akıner, Oguzhan Denizli, Osman Erganis

**Affiliations:** 1 Department of Microbiology, Faculty of Veterinary Medicine, Selcuk University, 42130 Konya, Turkey; zafersayin@gmail.com (Z.S.); aslisakmanoglu@gmail.com (A.B.); oguzhandenizli@outlook.com (O.D.); erganis@selcuk.edu.tr (O.E.); 2 Konya Veterinary Control and Research Institute, 42080 Konya, Turkey; yasin.gulcu@tarimorman.gov.tr (Y.G.); 213146001001@lisansustu.selcuk.edu.tr (I.A.); 3 Gözlü Agricultural Enterprise, 42430 Konya, Turkey; firatergen72@hotmail.com

**Keywords:** *Brucella abortus* S19, conjunctival, vaccine, long term screening

## Abstract

Brucellosis is still the most common zoonosis worldwide despite advanced technology and animal husbandry. Since there is still no effective *Brucella* vaccine for humans, it is crucial to control the disease in ruminants through eradication and vaccination. Although some countries around the world have achieved this circumstance, every country aims to become free of Brucellosis through vaccination, animal movements, and various eradication measures. For this purpose, the *Brucella abortus* S19 strain has been used safely for about 100 years. However, due to the O-polysaccharide (OPS) antigen in its structure, the antibody response created by the vaccine causes confusion in serological tests. For this purpose, researchers have provided both mucosal immunity and short-term antibody response by using the *B. abortus* S19 vaccine in conjunctival form instead of subcutaneous form. This study aimed to determine how long the post-vaccination titer levels persisted in animals vaccinated with vaccines from 3 different companies and different routes. In this study, a total of 115 calves aged 3 to 4.5 months were created in five groups, with 23 animals in each group: group 1 (vaccine brand A), group 2 (vaccine brand B), and group 3 (vaccine brand C) received the two-dose conjunctival vaccine, group 4 received the single-dose subcutaneous vaccine (vaccine brand C), and group 5 received the subcutaneous vaccine (vaccine brand C) plus the booster dose conjunctival vaccine (vaccine brand B). Brucellosis antibody titers were monitored each 21 days until the cattle were 26–28 months old. The collected sera were analyzed using the Rose Bengal Plate Test (RBPT), Serum Agglutination Test (SAT), and Complement Fixation Test (CFT), which are the preferred serological methods for Brucellosis eradication plans worldwide. In the conjunctival vaccination groups, only 3 (13%) of the animals in group 1 developed antibody titers one month after vaccination, and there was no antibody response detected against Brucellosis in group 2 and group 3. In animals that were stimulated conjunctivally, the threshold value of 30 International CFT Units (ICFTUs) (for distinguishing between infective titers and vaccination titers) was observed in one animal each in group 1 and group 2 and 0 animal in group 3. It was found that antibody titers turn to Brucellosis negative in all conjunctival vaccine groups at 7 months after vaccination. In groups 4 and 5, the first-month serological screening detected over 30 ICFTUs in 17 (89.47%) animals and 16 (69.5%) animals, respectively. In group 4, CFT titers were found to fall below 30 on the 17th month and 9.3 on the 22nd month. On the 14th month, the CFT titers of group 5 were found to be below 30, and all animals in this group turned negative after the 19th month. It was found that the single dose *B. abortus* S19 subcutaneous vaccination in calves caused persistent antibodies in 5% of the population. It is believed that persistent and high antibody titers created by subcutaneous vaccines will cause false positivity and create confusion in Brucellosis eradication programs. Therefore, although there is no clear distinction between vaccinated and infected animals, it has been observed that conjunctival Brucellosis vaccines create more stable antibody titers and decrease rapidly compared to subcutaneous vaccines. Based on the results of this study and the advantages of conjunctival vaccines, more effective eradication programs and antibody monitoring can be carried out in vaccinated herds where Brucellosis outbreaks are observed.

## Simple Summary

*Brucella* spp. is a bacteria that causes Brucellosis, a zoonotic disease that affects mammals, especially livestock. Since there is no effective vaccine for humans, controlling the disease in animals is vital. Although *B. abortus* S19, the first vaccine against bovine Brucellosis, cannot distinguish between the vaccinated and the infected like the vaccine strains studied in recent years, it is still used safely by various countries’ eradication programs. The *B. abortus* S19 vaccine was initially administered subcutaneously at high doses, but studies have shown that reduced doses provide similar protection when administered conjunctively. In this study, vaccines from three companies were administered to calves via conjunctival and subcutaneous routes, and long-term antibody changes were monitored to clarify the differences in antibody titers created by different routes of administration and to use these differences to advantage in detecting infected animals and in screening times. Findings show that antibody titers resulting from subcutaneous vaccination may cause confusion in the detection of positive animals. On the other hand, conjunctival vaccines can contribute to the rapid, permanent control of Brucellosis in cattle thanks to their short-term and low antibody titer formation. However, conjunctival vaccine should not be combined with subcutaneous vaccination in eradication plans.

## 1. Introduction

Brucellosis, which is of great importance for animal health, is classified as a neglected disease by the World Health Organization (WHO) [1]. Brucellosis has been reported as the most common zoonosis worldwide [2]. *Brucella abortus*, the causative agent of bovine Brucellosis, is characterized by abortions, infertility problems, calf losses, and reduced milk yield in dairy cows due to infection [3]. Infected animals shed large amounts of *B. abortus* via placenta, fetus, uterine discharge, and milk, thus posing a risk to farmers and veterinarians in terms of One Health [4]. Since there is still no effective *Brucella* vaccine for humans, it is essential to keep the disease under control through eradication and vaccination in animals (sheep, goats, cattle, pigs) [5,6]. *B. abortus* S19 vaccine strain was discovered in 1923 as an attenuated spot strain resulting from a 702-base erythritol catabolic gene deletion, has been used in the Mediterranean basin since 1941, and its protection was reported to be between 65–75% in the field [7,8,9]. Countries that used *B. abortus* S19 vaccine in Brucellosis eradication projects started to use conjunctival S19 vaccine due to the difficulty of administering subcutaneous *B. abortus* S19 vaccines, veterinarians being infected due to subcutaneous vaccines, confusion caused by vaccine-related seropositivity in serological tests in parenteral *Brucella* vaccination, rapid decrease in antibody levels in conjunctival immunization and provision of mucosal immunity [10].

The most practical and economical method for in-vivo disease screening is antibody screening tests (RBPT, SAT, CFT, etc.), but these tests also detect antibodies against LPS and O-polysaccharide (OPS) antigens, which are the immunodominant surface antigens of smooth *Brucella* spp. [11,12]. The OPS in the *Brucella* LPS structure provides antigenically protective activity, but its presence results in similar antibody profiles in infected and vaccinated animals [13,14]. Differentiating Infected from Vaccinated Animals (DIVA) vaccines have attempted to overcome this problem by developing OPS-free vaccines. These include protein subunit, DNA, and vectored vaccines [10], but the currently only licensed vaccine is the rough *B. abortus* RB51, which enables the differentiation between the vaccinated and the infected in mass vaccination for *Brucella* eradication plans in many countries [15]. However, in most countries, the S19 vaccine strain is still used as a conjunctival and subcutaneous form [16]. In ideal Brucellosis vaccination, a Th1 response is desired, and long-term antibody titers are not desired; however, it has been reported that antibodies against subcutaneous injection vaccines cause longer-term antibodies than conjunctival vaccines [17]. In infected animals, higher and longer-lasting antibodies are formed than antibodies formed by vaccines [10]. Since Brucellosis is an intracellular bacterium, it should not be assumed that high antibody titers will provide protective immunity against the disease in vaccination; model studies in mice have reported that cellular and humoral responses provide protection separately [18]. When comparing 19 countries in terms of their bovine Brucellosis control program, it was reported that 12 countries used the S19 vaccine, and the RB51 vaccine was used by seven countries. Reduced dose S19 vaccines have been reported to be used in adult cattle and total dose subcutaneous or conjunctival vaccination programs in calves. In terms of serological testing of Brucellosis, among 19 countries and regions, RBPT was used as a screening test by 12 countries, while the SAT was reported to be used by five countries. While the gold standard CFT is used by 14 countries mainly as a confirmatory test, it has been reported that the ELISA test is used in the surveillance programs of four countries using the RB51 vaccine [16,19].

In eradication plans where *B. abortus* S19 is used subcutaneous/conjunctival, the titers of vaccinated and infected animals cannot be separated, and positive animals cause confusion. This process needs to be clarified to prepare more effective eradication plans. The study aimed to determine the duration of change in titer levels using SA and CF tests in animals vaccinated with three different commercially available vaccines via different stimulation routes.

## 2. Materials and Methods

### 2.1. Vaccination and Sample Collection

**Figure 1:**
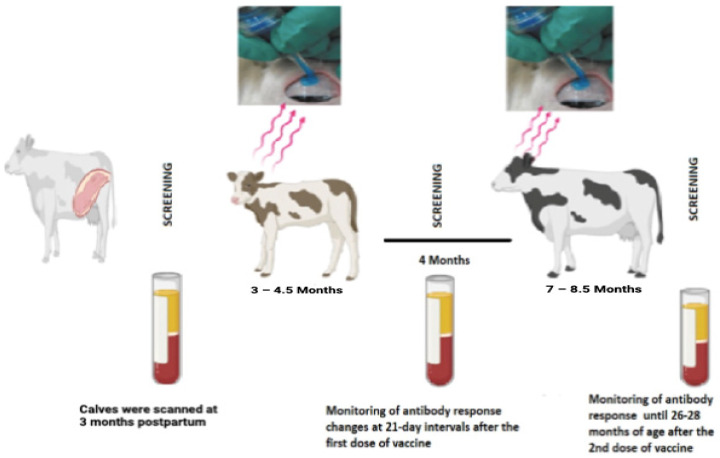
Study hypothesis and working plan.

The study was carried out on a dairy farm in Konya, Turkey (37°52′ N, 32°29′ E) between December 2021 and December 2023. This Brucellosis-free farm, which has 3100 Brown Swiss and Simmental cattle, was screened for Brucellosis, and all were found negative. Calves born between August and October 2021, aged 3 months, were screened for Brucellosis during the pre-vaccination period (after maternal antibody levels had decreased). One hundred fifteen calves aged between 3 and 4.5 months were vaccinated by dividing them into five groups according to the study groups below. In the vaccine groups with two dose applications, the second dose of vaccine was administered 4 months after the first dose of vaccine (aged 7–8.5 months) (Figure 1). The study was conducted in collaboration with three GMP-certified organizations in Turkey that produce *Brucella* vaccines, one state-owned and the other two private-sector [20]. Vaccines belonging to the vaccine companies used in the study were numbered A, B, and C due to ethical concerns (Table 1). Since the study aimed to monitor antibody levels of different *Brucella* vaccinations, which is mandatory by regulation, an unvaccinated group was not added. Vaccination groups were organized according to the vaccines and vaccination routes allowed by the regulations in the field.

### 2.2. Serological Tests

During the screening, blood was collected from the animals using a sterile needle from the jugular vein and filtered into tubes without anticoagulant. The blood tubes were delivered to Selcuk University, Faculty of Veterinary Medicine, Microbiology Laboratory, in the cold chain. The blood tubes were centrifuged at 5000 rpm × 5 min to separate the sera. The sera were first tested with the RBPT. For RBPT (Seromed A.Ş, Istanbul, Turkey), 25 µL of test antigen was mixed with an equal amount of calf serum on a slide, which was rotated for four min. For SAT, a fixed amount of suspicious calf serum from SAT antigen prepared from *B. abortus* S99 antigen (SAT Antigen, Vetal A.Ş, Adiyaman, Turkey) was added to the wells of a 96-well U-bottom plate starting from 1/10 dilution ratio up to 1/1280 dilution ratio and left for incubation at 37 °C overnight. The wells with antigen–antibody agglutinations were examined. For CFT, diluted serums were distributed to the wells of the plates. A fixed amount of antigen (SN 437, IDEXX, Montpellier, France) and veronal buffer (KM0030, Virion-Serion GmbH, Würzburg, Germany) were added to the wells. Antigen-serum mixture and control wells were incubated at 37 °C for 30 min. Then, a complement (KM0038, Virion-Serion GmbH, Würzburg, Germany) was distributed to each well and incubated at 37 °C for 30 min; after this step, amboceptor and Sheep Red Blood Cell (SRBC) were also distributed to each well. Plates were incubated at 37 °C for 30 min and incubated at 4 °C for 2 h to settle the non-lysed cells. The results were 100% hemolysis inhibition: 4 ++++, 75% hemolysis inhibition: 3 +++, 50% hemolysis inhibition: 2 ++, 25% hemolysis inhibition: 1 + were evaluated. The test result is given to show the ICFTU in each ml of the tested serum. The formula ICFTU_TESTSERUM_ = F × 1/T_USAbS_ was used to calculate the ICFTU value. According to the CF test, a titer of ICFTU 30 and above was considered positive, and in the SA test, a titer of 1/20 and above was considered positive. The lowest detectable titer, ICFTU 5, was used as another threshold value to measure the sensitivity of the CF test. All tests were completed by referring to the Diagnostic Method Union book of the Ministry of Agriculture and Forestry of the Republic of Turkey [21].

### 2.3. Statistical Analysis

The data were evaluated in the statistical package program IBM SPSS Statistics Standard Concurrent User V 265 (IBM Corp., Armonk, NY, USA) and the Minitab program. Descriptive statistics were given as unit $\bar{x}$ + ss mean ± standard deviation, median (M), and interquartile range (IQR) values. The distribution of data belonging to the scales was evaluated using the Shapiro-Wilk normality test. When the number of groups was more than two, they were compared using the Kruskal-Wallis H test. The Bonferroni Dunn post hoc method was used for multiple comparisons. Proportional differences between groups according to months were determined using the “Two Proportion Z Test.” Chi-squared tests were used to compare categorical variables with each other. In all comparisons, *p* < 0.05 was considered statistically significant.

## 3. Results

The study started with 23 animals in each group, but animal losses occurred in some groups during the study: group 1: 22 (one animal died after the first month), group 2: 23, group 3: 23, group 4: 19 (one animal died in the 5th month and one in the 18th month, two animals died at in 19 months), group 5: 23.

When the first vaccination was completed, the oldest animal in the groups was recorded as 4.5 months old, and the youngest animal was recorded as 3 months old. In the second vaccination, these animals were recorded as 8.5 months old and 7 months old, respectively. When the study was completed, the age ranges of the animals were between 26–28 months. The age differences between the oldest and youngest calves in the vaccination groups were determined as 12 days in group 1, 9 days in group 2, 12 days in group 3, 14 days in group 4, and 7 days in group 5. No statistically significant difference in age was found between the groups.

Of the conjunctival vaccine groups, no animal in group three developed a CFT titer over 30. In one calf each from group 1 and group 2, the titer was observed to exceed 30 for a short time at the sixth (44.2 ICFTU) and second (52.6 ICFTU) months, respectively, and then the titer disappeared. One month after conjunctival vaccination, only three (13%) of the animals in group 1 showed antibody titers, while no antibody response was observed in groups 2 and 3. In group 4 and group 5, in the first month after the first vaccination, CFT titers over 30 were detected in 17 (89.47%) animals and 16 (69.5%) animals, respectively (Table 2). A statistically significant difference was found between the conjunctival vaccine group and the subcutaneous vaccine groups in terms of the number of seropositive animals in the vaccine groups (*p* < 0.05). No statistical difference was found between group 4 and group 5.

**Figure 2:**
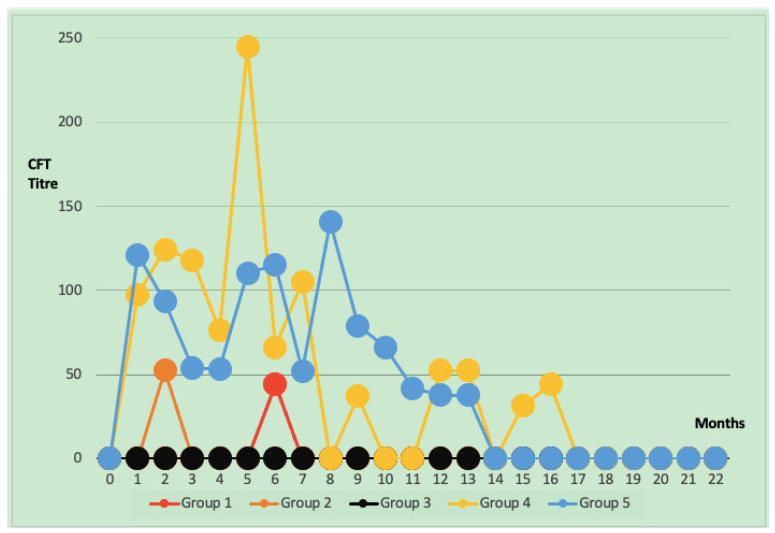
Distribution of mean titer values of animals with ICFTU >30 at different periods after vaccination.

After vaccination, SAT and CFT titers in conjunctival vaccine groups were found to have short-term increases and sudden decreases. In all groups that received conjunctival vaccination at the 7th month, antibody titers against Brucellosis in the CF test were negative (below the threshold value of 30 units), and no titers could be detected after the 10th month. In group 4, CFT titers were found to be negative below the threshold value of 30 titers after the 17th month (Figure 2). The CFTU5 and ICFTU30 values are statistically different between conjunctival and subcutaneous groups *p* < 0.05. Group 4 and group 5 values are statistically higher than values in groups 1, 2, and 3 (Table 3).

**Figure 3:**
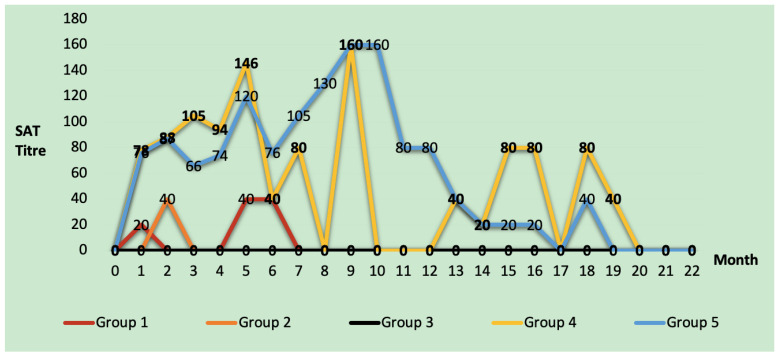
Distribution of mean titer values of animals with SAT > 1/20 in different periods after vaccination.

According to the SAT results, animals in group 1 and group 2 had a titer of 1/40, but no animal in group 3 ever reached this titer. In these three vaccine groups, antibody titers were observed to decrease rapidly within 4 months after the first vaccine dose until the booster dose. In group 4, some animals had positive titers in the CF test for up to 17 months and the SA test for up to 20 months after the application. Group 5 had 30 ICFTU titers up to 14 months after application and 1/40 titers up to 19 months in the SAT (Figure 3). The number of SAT-positive animals in the first month was 21 in groups 4 and 5. This number decreased by 85.7% to 3 in group 4 and by 71.4% to 6 in group 5 in the fourth month. In group 4, three different animals with SAT titers of 1/160 and above were found in the fourth month. In group 5, five animals with a titer of 1/160 were detected up to the 10th month, and 1/320 SAT titer was detected in different animals in the fourth, eighth, and ninth months (Table 4).

In group 4, It was determined that the CFT titer reached 1000 and SAT titer 1/320 in the fifth month. It was determined that the titer of this animal, whose CFT titer reached 1000, turned negative in the eighth month, like all other animals in the group, but started to increase by following a fluctuating course from the 11th month. The titer dropped below 30 in the 17th month and remained at 9.3 until the end of the study. In group 5, CFT titers dropped below 30 in the 14th month, and the entire group population was negative in the 19th month. It was found that titers fluctuated and persisted in this manner in 5% of the population in group 4. In group 5, the percentage of animals with fluctuating long-term antibody titers in the population was determined as 8.7%.

When the results of the subcutaneous vaccine groups with the highest seropositive titers were compared using serological tests, it was determined that CFT, the gold standard for *Brucella* diagnosis, had a statistically higher sensitivity than SAT in the third month in group 4 (*p* < 0.05). No statistically significant difference was found between the sensitivities of both tests in detecting positive animals in the groups in the other months in group 4 and until the 22nd month in group 5 (Table 5).

## 4. Discussion

In this study, calves vaccinated with different *Brucella* vaccines available on the market in Turkey using different methods were monitored from birth to 2.5 years of age, and post-vaccination titer changes were observed. Since the current vaccine efficacy test and registration studies are based on challenge potency studies conducted on model animals such as mice and target animals at 1-month post-vaccination, data on longer-term seropotens are more limited [22,23,24]. In challenge trials of subcutaneous *B. abortus* S19 vaccinated animals, it was reported that 70–91% of the cattle were protected from abortion [25,26]. The most significant question mark regarding the *B. abortus* S19 vaccine is the reported cases of abortion it caused in pregnant cattle when administered subcutaneously [27]. It has been reported that subcutaneous vaccinations cause 3.2% of abortions, while intravenous vaccinations cause 100% of abortions [28,29,30]. Plomment and Plomment (1975) and Fensterbank and Plomment (1979) subsequently conducted a series of efficacy studies in the model animal and target animal cattle. They demonstrated that conjunctival vaccination provides similar protection to injectable vaccination against Brucellosis [22,31,32,33,34,35]. An ideal vaccine against Brucellosis should have the following properties: (i) be live and capable of inducing a strong T helper type 1 immune response (Th1); (ii) not induce antibodies that interfere with serological tests used to diagnose infected cattle, regardless of the route of administration, dose, age or sex of the animals; (iii) be attenuated and not cause disease or persistent infection in immunized animals and should not be pathogenic to humans; (iv) be able to provide strong and long-lasting protection against systemic and uterine infections, as well as preventing abortions in pregnant animals vaccinated with a single dose; (v) not lead to seroconversion on revaccination; (vi) be stable and not revert virulence in vivo or in vitro; and (vii) be inexpensive, easy to manufacture and administer [36,37,38,39]. According to the FAO (2003) animal Brucellosis surveillance program, it was reported that 80% of the population should be positive in the serological test to be performed from blood samples collected randomly during the second and third weeks after subcutaneous immunization [40]. In the current study, when SAT-positive animals were examined, the number of animals with titer higher than 1/20 was determined as 91.3% in groups 4 and 5. In the conjunctival vaccine groups, no animals were detected to have titers in the RBP, SA, and CF tests at week 3 after vaccination.

In the study conducted by Plommet and Plommet (1976), animals vaccinated by conjunctival and subcutaneous routes were observed for 61 weeks until the gestational age. Seropositivity was detected for 58 weeks in the single-dose subcutaneous vaccine group and 61 weeks in the first-dose subcutaneous booster-dose conjunctival vaccine group. However, seropositivity was not as high in the conjunctival vaccine group as in the injectable group, and seropositivity was detected for 44 weeks only in 3 animals in the group [33]. Fensterbank and Plommet (1979) reported that antibody titers persisted for 58 weeks in animals given the first dose of subcutaneous S19, followed by the second dose of conjunctival vaccine. In contrast, titers were negative in 12 weeks in animals given only two doses of conjunctival vaccine [34]. However, this study observed that titers became negative after the seventh month in three different conjunctival vaccine groups (groups 1, 2, and 3). Brucellosis titers of animals in group 4 fell below 30 ICFTU in the 17th month and 9.3 ICFTU in the 22nd month. In group 5, the titers were below 30 ICFTU in the 14th month, and the titers of all groups were negative after the 19th month. Plommet and Plommet (1976) conducted a study whose results for the subcutaneous vaccine groups were similar to those of our research. However, while Plommet and Plommet (1976) found positivity up to 11 months in the booster-dose conjunctival vaccine groups [33], in our study, the titers of the animals turned negative in the conjunctival groups in the seventh month. It is thought that this difference may be due to the different ages.

In Brazil, when a single dose of S19 subcutaneous was administered to adult Angus (3–5 years old), most of the population was reported to be seronegative after 9 months [41]. In a trial in which a single dose of S19 was administered, it was reported that titers continued for 4 months in adult cattle and 2 months in calves [42]. In another study, 230 heifers aged 3–8 months from 11 farms were serologically monitored at 1-month intervals for 27 months after S19 vaccination. Three animals were found to be RBPT positive at 23 months, and one animal was found to be CFT positive at 12 months. It was reported that there were more positive animals vaccinated at 15 months of age [43]. In a conjunctival vaccine surveillance study conducted in India, 366 *Brucella*-free cattle were vaccinated conjunctively with the reduced-dose *B. abortus* S19 vaccine, and all were reported to be serologically negative by RBPT at 12 weeks [44]. In a study conducted in South Africa, it was reported that 58 heifers aged 3–12 months and negative for RBPT were vaccinated subcutaneously at a dose of 5 × 10^10^, and 12% were detected serologically positive according to serological screening after 4.5 years [45]. In a study comparing the cellular immunity parameters of *B. abortus* S19 and RB51 vaccines through day 575, they reported that the number of CD8+ T cells decreased after day 210 compared to day 28 but was significantly higher than day 0 until day 575 [36]. In another conjunctival vaccine efficacy study conducted in Turkey, animals aged 3 months to 11 years were serologically monitored for 7 months. Researchers reported that in the vaccinated groups, antibody titers in 3–8 month-old animals decreased from the second month onwards, while in older animals, the titers continued even at the seventh month. It has been reported that the number of positive animals in the population increases as the age of the animal increases [46]. In the S19 vaccine trial conducted with 252 adult animals, 3–5 months pregnant, from 3 separate herds free of *Brucella*, at a reduced dose of 3 × 10^8^ CFU/dose, the animals were observed for 4 years. It was determined that the antibody titers of the animals were above 6.75% in the first year, 4% in the second year, 4.5% in the third year, and 0.82% in the fourth year. As a result of the study, the researchers reported that the administration of subcutaneous vaccines to adult animals, even at a reduced dose, would cause permanent titers [47]. In this study, the first dose of vaccination was administered to calves aged 3–4.5 months, and the booster dose was administered 4 months later when the calves were 7–8.5 months old. Additionally, no statistically significant age difference was found between the vaccine groups. Compared with other study results, the antibody response to subcutaneous S19 vaccines appears to be higher. It lasts longer as the administered dose (CFU/mL) increases and the age of the animals increases. In this study, it was determined that there were animals that had titers for 19–22 months in the groups that were applied subcutaneous immunization at 40–120 × 10^9^ CFU/dose.

In countries where the S19 vaccine strain is used, the highly sensitive RBP test is first used for pre-field screening. High-specificity tests like the SAT and CF test (Gold standard) are performed to prevent false positives and false negatives and to detect positive animals ideally [3]. The sensitivity and specificity of the SA and CF tests are subjects of comparison in serological Brucellosis studies [48]. In this study, since the number of animals with titers in the host groups was minimal, the statistical comparison could not be made in terms of the sensitivity of serological tests. In our study, in the comparison study conducted in the first 4 months when positivity was most common in the subcutaneous vaccine groups. It was determined that the CF test detected more positive animals than the SAT only in the group 4 in the 3rd month. However, in other months, no statistical difference was found in the power to detect positive animals between CF and SA tests.

According to the FAO surveillance study, if the cattle herd is vaccinated with the subcutaneous vaccine, diagnosis can be made by serological test 12 months after the last vaccination [40]. The European Union cattle, sheep, and goat Brucellosis eradication plan is reported in the Animal Health Law/429/2016 regulation as follows: if the herd has been vaccinated, sampling is performed at 6–12 month intervals after vaccination, depending on the route of administration of the vaccine [39]. In other countries’ bovine Brucellosis control programs using the *B. abortus* S19 vaccine strain, there is a statement that if there is no abortion in the vaccinated animals in positive herds, blood samples should be taken after 12 months to avoid confusion. According to the United States Department of Agriculture (USDA) Bovine Brucellosis regulations, if a Brucellosis-positive herd is included in the vaccination program (with the *B. abortus* RB51 vaccine without OPS), the herd should be screened for 18 months plus 12 months (total 30 months) in 6-month periods until the titers turn negative [49]. Although USDA does not use the S19 vaccine strain, it ensures the end of the disease. It prevents disease spread by screening the herd at 6-month intervals for at least 18 months and distinguishing false-positive and positive animals within the herd. According to the results of the study, it has been observed that increasing the first screening time from 12 to 18 months in positive herds in countries that use subcutaneous S19 vaccine in Bovine Brucellosis eradication programs prevents seropositivity development due to the vaccine. In addition, the study results determined that the conjunctival vaccine groups with low antibody titers and rapid decreases in this titer allowed the distinction between infected and vaccinated animals in earlier months compared to the subcutaneous S19 vaccine groups.

Also, according to the study results, it was observed that 5–8.7% of the population administered subcutaneous vaccine caused fluctuating and long antibody titers. It has been determined that the animals that had positive titers due to the vaccine, which constitute 5–8.7% of the population, will be eradicated unnecessarily and will cause severe economic losses. In addition, it is thought that intramuscular application errors during the injection of the vaccine, injection of an excessive dose during the vaccine administration, and adult vaccination will cause the titer to continue longer.

## 5. Conclusions

According to the study results, even when conjunctival *B. abortus* S19 vaccines from three different vaccine companies were used, antibody titers rapidly decreased. Conjunctival use of the *B. abortus* S19 vaccine would help distinguish between the infected and the vaccinated within the herd after 7 months. It was also resolved what percentage of animal populations the vaccines induced a serological response. In eradication programs where the *B. abortus* S19 vaccine is used in combination with conjunctival and subcutaneous forms, attention should be paid to persistent antibody titers of the subcutaneous vaccine. This circumstance overrides the advantages of conjunctival vaccination; therefore, if possible, the subcutaneous vaccine should not be added to eradication programs where conjunctival vaccination is performed, or expert *Brucella* epidemiologists should continuously monitor herd antibody titers.

## Figures and Tables

**Table 1:** Detailed status of working groups.

Groups	Number of Animals	Vaccine Company	Dose Number	Route of Vaccine Application	Number of S19 Bacteria in One Dose of Vaccine
Group 1	23	A	Two dose	Conjunctival	5–10 × 10^9^ CFU/dose
Group 2	23	B	Two dose	Conjunctival	5–10 × 10^9^ CFU/dose
Group 3	23	C	Two dose	Conjunctival	5–10 × 10^9^ CFU/dose
Group 4	23	C	Single dose	Subcutaneous	40–120 × 10^9^ CFU/dose
Group 5	23	C	Two dose	Subcutaneous (1. Dose)	40–120 × 10^9^ CFU/dose
		B		Conjunctival (2. Dose)	5–10 × 10^9^ CFU/dose

**Table 2:** Number of animals with SAT > 1/20, ICFTU > 5, ICFTU > 30 ^a^ titers at different periods of vaccination *.

	Group 1			Group 2			Group 3			Group 4			Group 5		
Months	SAT > 1/20	ICFTU > 5	ICFTU > 30	SAT > 1/20	ICFTU > 5	ICFTU > 30	SAT > 1/20	ICFTU > 5	ICFTU > 30	SAT > 1/20	ICFTU > 5	ICFTU > 30	SAT > 1/20	ICFTU > 5	ICFTU > 30
0	0	0	0	0	0	0	0	0	0	0	0	0	0	0	0
1	3 ^β^	2 ^y^	0 ^b^	0 ^β^	0 ^y^	0 ^b^	0 ^β^	0 ^y^	0 ^b^	21 ^α^	22 ^x^	17 ^a^	21 ^α^	21 ^x^	16 ^a^
2	0 ^β^	1 ^y^	0 ^b^	1 ^β^	1 ^y^	1 ^b^	0 ^β^	2 ^y^	0 ^b^	14 ^α^	17 ^x^	12 ^a^	14 ^α^	18 ^x^	13 ^a^
3	0 ^β^	1 ^y^	0 ^b^	0 ^β^	0 ^y^	0 ^b^	0 ^β^	0 ^y^	0 ^b^	8 ^α^	17 ^x^	9 ^a^	12 ^α^	17 ^x^	9 ^a^
4	0 ^β^	0 ^y^	0 ^b^	0 ^β^	0 ^y^	0 ^b^	0 ^β^	0 ^y^	0 ^b^	10 ^α^	12 ^x^	5 ^a^	11 ^α^	10 ^x^	5 ^a^
5	1 ^β^	3 ^y, z^	0 ^b^	0 ^β^	2 ^z^	0 ^b^	0 ^β^	1 ^z^	0 ^b^	3 ^α, β^	7 ^x, y^	5 ^a^	6 ^α^	10 ^x^	5 ^a^
6	1 ^α, β^	2 ^x, y, z^	1 ^a^	0 ^β^	0 ^z^	0 ^a^	0 ^β^	0 ^z^	0 ^a^	3 ^α^	5 ^x^	2 ^a^	5 ^α^	5 ^x, y^	2 ^a^
7	1 ^α, β^	0 ^y^	0 ^a^	0 ^β^	0 ^y^	0 ^a^	0 ^β^	1 ^y^	0 ^a^	5 ^α^	4 ^x^	1 ^a^	4 ^α^	3 ^x, y^	3 ^a^
8	0 ^α^	0 ^x^	0 ^a^	0 ^α^	0 ^x^	0 ^a^	0 ^α^	0 ^x^	0 ^a^	0 ^α^	0 ^x^	0 ^a^	3 ^α^	3 ^x^	2 ^a^
9	0 ^β^	1 ^x^	0 ^a^	0 ^β^	0 ^x^	0 ^a^	0 ^β^	0 ^x^	0 ^a^	1 ^α, β^	1 ^x^	1 ^a^	5 ^α^	2 ^x^	2 ^a^
10	0 ^α^	0 ^x^	0 ^a^	0 ^α^	0 ^x^	0 ^a^	0 ^α^	0 ^x^	0 ^a^	0 ^α^	0 ^x^	0 ^a^	2 ^α^	2 ^x^	2 ^a^
11	0 ^α^	0 ^x^	0 ^a^	0 ^α^	0 ^x^	0 ^a^	0 ^α^	0 ^x^	0 ^a^	0 ^α^	1 ^x^	0 ^a^	2 ^α^	2 ^x^	2 ^a^
12	0 ^α^	0 ^x^	0 ^a^	0 ^α^	0 ^x^	0 ^a^	0 ^α^	0 ^x^	0 ^a^	0 ^α^	1 ^x^	1 ^a^	2 ^α^	2 ^x^	2 ^a^
13	0 ^α^	0 ^x^	0 ^a^	0 ^α^	0 ^x^	0 ^a^	0 ^α^	0 ^x^	0 ^a^	1 ^α^	1 ^x^	1 ^a^	2 ^α^	2 ^x^	2 ^a^
14	0 ^α^	0 ^x^	0 ^a^	0 ^α^	0 ^x^	0 ^a^	0 ^α^	0 ^x^	0 ^a^	1 ^α^	1 ^x^	0 ^a^	2 ^α^	2 ^x^	0 ^a^
15	0 ^α^	0 ^x^	0 ^a^	0 ^α^	0 ^x^	0 ^a^	0 ^α^	0 ^x^	0 ^a^	1 ^α^	1 ^x^	1 ^a^	1 ^α^	0 ^x^	0 ^a^
16	0 ^α^	0 ^x^	0 ^a^	0 ^α^	0 ^x^	0 ^a^	0 ^α^	0 ^x^	0 ^a^	1 ^α^	1 ^x^	1 ^a^	1 ^α^	2 ^x^	0 ^a^
17	0 ^α^	0 ^x^	0 ^a^	0 ^α^	0 ^x^	0 ^a^	0 ^α^	0 ^x^	0 ^a^	0 ^α^	2 ^x^	0 ^a^	0 ^α^	2 ^x^	0 ^a^
18	0 ^α^	0 ^x^	0 ^a^	0 ^α^	0 ^x^	0 ^a^	0 ^α^	0 ^x^	0 ^a^	1 ^α^	1 ^x^	0 ^a^	1 ^α^	1 ^x^	0 ^a^
19	0 ^α^	0 ^x^	0 ^a^	0 ^α^	0 ^x^	0 ^a^	0 ^α^	0 ^x^	0 ^a^	1 ^α^	1 ^x^	0 ^a^	0 ^α^	0 ^x^	0 ^a^
20	0 ^α^	0 ^x^	0 ^a^	0 ^α^	0 ^x^	0 ^a^	0 ^α^	0 ^x^	0 ^a^	0 ^α^	1 ^x^	0 ^a^	0 ^α^	0 ^x^	0 ^a^
21	0 ^α^	0 ^x^	0 ^a^	0 ^α^	0 ^x^	0 ^a^	0 ^α^	0 ^x^	0 ^a^	0 ^α^	1 ^x^	0 ^a^	0 ^α^	0 ^x^	0 ^a^
22	0 ^α^	0 ^x^	0 ^a^	0 ^α^	0 ^x^	0 ^a^	0 ^α^	0 ^x^	0 ^a^	0 ^α^	1 ^x^	0 ^a^	0 ^α^	0 ^x^	0 ^a^

* The letters α and β in the same row represent differences between values greater than SAT 1/20 and are statistically significant (*p* < 0.05, Chi-square). The letters x, y, and z in the same row represent differences between values greater than ICFTU 5 and are statistically significant (*p* < 0.05, Chi-square). The letters a and b in the same row represent differences between values greater than ICFTU 30 and are statistically significant (*p* < 0.05, Chi-square).

**Table 3:** Distribution of mean CFT titer values of positive animals at different periods after vaccination.

CFT Titer	Groups					Test Statistics	
	1	2	3	4	5	*H* Value	*p* Value
ICFTU 5	3.16 ± 7.19 0 (5.31) ^a^	2.95 ± 11.40 0 (0) ^a^	1.47 ± 4.08 0 (0) ^a^	40.19 ± 39.91 32.93 (58.41) ^b^	37.45 ± 32.05 36.92 (58.41) ^b^	58.303	<0.001
ICFTU 30	2.01 ± 9.42 0 (0) ^a^	2.39 ± 11.21 0 (0) ^a^	0.0 ± 0.0 0 (0) ^a^	47.72 ± 61.63 34.22 (81.41) ^b^	45.54 ± 47.09 39.82 (82.50) ^b^	41.233	<0.001

Values are given as mean ± standard deviation and median (interquartile range). H: Kruskal Wallis test, superscripts a and b represent statistically different groups.

**Table 4:** Periods in the study when the highest antibody titers occurred after vaccination.

Vaccine Groups	SAT	ICFTU	Month of Sampling
Group 1 (conjunctival)	1/40	44.2	6. Month
Group 2 (conjunctival)	1/40	52.6	2. Month
Group 3 (conjunctival)	0	15.6	5. Month
Group 4 (subcutaneous)	1/320	1000	5. Month
Group 5 (subcutaneous + conjunctival)	1/160	353	1. Month
Group 5 (subcutaneous + conjunctival)	1/320	250	8. Month

**Table 5:** Differences in detecting positive animals on SAT and CF tests in group 4 and group 5.

Months	Number of Positive Animals in Group 4			Number of Positive Animals in Group 5		
	SAT > 1/20	ICFTU > 5	*p*	SAT > 1/20	ICFTU > 5	*p*
1	21	22	0.549	21	21	0.999
2	14	17	0.341	14	18	0.192
3	8	17	0.004 *	12	17	0.117
4	10	12	0.553	11	10	0.767

* Two Proportion Z Tests were applied, and in group 4, there was a statistically significant difference in the third month. The break occurred in the third month. In group 5, the values obtained in the Two Proportion Z Test on the numbers of animals found positive according to the CFT and SAT are statistically similar according to the months.

## Data Availability

The original contributions presented in this study are included in the article. Further inquiries can be directed to the corresponding author.
